# “Very” Very Late Stent Thrombosis: The Occurrence of Thrombosis 12.3 Years After Paclitaxel-Eluting Stent Implantation

**DOI:** 10.7759/cureus.53010

**Published:** 2024-01-26

**Authors:** Hung D Tran, Ha V. T Pham, Thang D Vu

**Affiliations:** 1 Cardiovascular Center, Hospital 103/Vietnam Military Medical University, Hanoi, VNM

**Keywords:** stent thrombosis, paclitaxel eluting stent, neointima, neoatherosclerosis, drug-eluting stents, coronary artery disease

## Abstract

Very late stent thrombosis (VLST) refers to stent thrombosis occurring beyond one year after coronary intervention. “Very” very or extremely late stent thrombosis (VVLST), occurring after five years of drug-eluting stent (DES) implantation, is extremely rare. We report a case of a 60-year-old male patient with ST-elevation myocardial infarction (STEMI) due to in-stent thrombosis 12.3 years after first-generation DES implantation; we also engage in a brief discussion of its pathogenesis and prevention.

## Introduction

In-stent thrombosis is a rare complication after coronary stent implantation, with a reported annual incidence of 0.4-0.6% for stent placement beyond one year [1]. Stent thrombosis after one year of placement is classified as very late stent thrombosis (VLST), a potentially life-threatening complication that is more commonly seen with drug-eluting stent (DES), and usually presents as acute myocardial infarction (MI) or sudden death [2]. This occurs more frequently with first-generation DES than bare metal stents (BMS) and the majority of VLST cases happen within four years after stent implantation [3]. Stent thrombosis after five years of implantation is extremely rare, and has been referred to as ‘‘very” very or extremely late stent thrombosis (VVLST). Current smoking, a long stent, and non-compliance with medical therapy have been suggested as risk factors that may lead to defects in the neointima and neoatherosclerosis formation in the stent, and predispose it to thrombotic events [4,5]. However, the pathophysiology of VVLST is not fully understood due to the lack of sufficient evidence, especially in Vietnamese patients. We present the first reported case of VVLST in Vietnam presenting as ST-elevated myocardial infarction (STEMI) 12.3 years after first-generation DES implantation.

## Case presentation

A 60-year-old male, a smoker with no medical history, presented to our hospital with a right ventricular myocardial infarction on August 27, 2010. He underwent percutaneous coronary intervention (PCI) to the right coronary artery (RCA) using one TAXUS® LiberteTM paclitaxel-eluting stent (2.75 x 32 mm at 16 atm). He was discharged home on dual antiplatelets (aspirin 100 mg, clopidogrel 75 mg) and atorvastatin 20 mg. Aspirin and clopidogrel were discontinued after 12 months.

**Figure 1:**
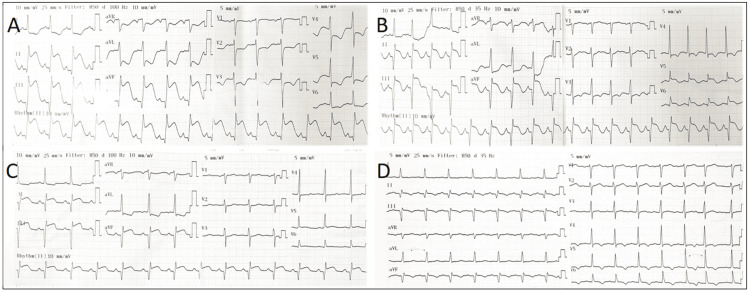
Electrocardiogram of the patient (A) On admission. (B) After thrombosuction and balloon angioplasty of RCA. (C) After PCI (on the ninth day). (D) After 12 months PCI: percutaneous coronary intervention; RCA: right coronary artery

**Figure 2:**
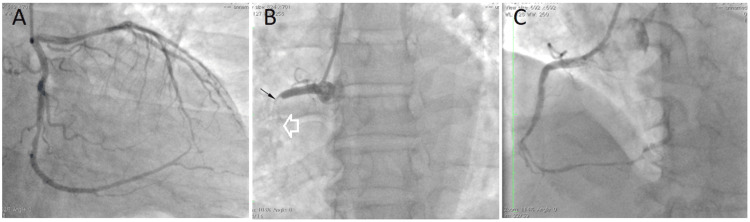
Coronary angiogram of the patient A) Nonsignificant lesion of LAD and LCx (<50% stenosis as measured with QCA). (B) Proximal complete occlusion of RCA (small arrow) due to thrombus in the stent (white arrow). (C) The RCA after thrombosuction and balloon angioplasty with restored TIMI 2 flow and diffuse lesions LAD: left anterior descending coronary artery; LCx: left circumflex coronary artery; QCA: quantitative coronary angiography; RCA: right coronary artery; TIMI: Thrombolysis in Myocardial Infarction

The patient remained asymptomatic without antiplatelets until November 19, 2022 (12.3 years after stent implantation), when he presented complaining of chest heaviness one hour prior, and ECG showed ST elevation in lead II, III, aVF, and ST depression in V2-V6, lead I, aVL (Figure 1A). The troponin-T level was elevated. Two-dimensional (2D) echocardiography showed hypokinesis of the inferior wall and a left ventricular ejection fraction (LVEF) of 45%. His emergency coronary angiogram showed acute occlusion of proximal RCA with a moderate thrombus burden (Figure 2B) and nonsignificant lesions of other vessels (Figure 2A).

**Figure 3:**
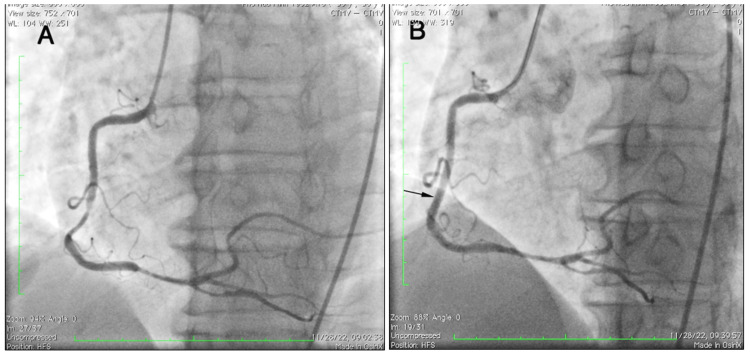
Stent placement (A) RCA before stent placement. (B) Stent placement in the mid-RCA (black arrow) RCA: right coronary artery

The patient was given oral ticagrelor 180 mg, aspirin 325 mg, and a single intravenous bolus of 30 mg, followed in 15 minutes by a subcutaneous injection of enoxaparin (1 mg/kg, every 12 hours) before PCI. After thrombosuction and balloon angioplasty, Thrombolysis in Myocardial Infarction (TIMI) 2 flow was restored, and diffuse lesions in RCA were observed (Figure 2C). The door-to-balloon time was four hours. Since ECG and echocardiography showed the culprit lesion in RCA and the patient was hemodynamically stable, we decided to stage RCA intervention with the continuation of antithrombotic drugs (enoxaparin, ticagrelor, and aspirin) before the second coronary intervention. A repeated coronary angiogram after nine days on antithrombotic drugs revealed less in-stent thrombus and only one significant lesion in mid-RCA (Figure 3A). The RCA was stented with one second-generation everolimus-eluting stent (2.75 x 23 mm) (Figure 3B). The LVEF after the procedure was 45%. The patient was discharged after 14 days and was prescribed daily aspirin 100 mg, ticagrelor 180 mg, metoprolol 50 mg, and atorvastatin 20 mg. He remained well during regular follow-ups in the next 12 months, with good effort tolerance and no angina.

## Discussion

Stent thrombosis after five years of stent implantation is extremely rare, and this condition has been termed VVLST. In 2009, Layland et al. reported the first case of VVLST in a patient after 2037 days of deployment [6]. In 2011, Taylor Sutton et al. reported another case of VVLST occurring five years after implantation [7]. In 2014, Kaliyadan et al. published a paper involving seven patients with VVLST after 5.6-7.1 years of stent implantation [8]. Recently, Sleiman et al. (2020) and Kumar et al. (2022) reported cases of VVLST 12 years after receiving first-generation paclitaxel-eluting stents [9]. Since the initial approval of the first generation of DES for clinical use in 2003, millions of patients worldwide with coronary diseases have benefited from this technology [10]. However, DES implantation is associated with delayed endothelialization, leading to exposed stent struts, stent malapposition, and an increased risk of early and late in-stent thrombosis, especially with first-generation DES.

In-stent neoatherosclerosis has been reported in 30% of VLST cases at autopsy and is considered the most significant risk factor of VLST pathogenesis post-DES implantation [11]. The mechanism underlying the development of neoatherosclerosis is not entirely understood and could be associated with functional immaturity of the intimal endothelium, neointimal erosion, plaque rupture, hypersensitivity reactions, uncovered struts, overlapping, and malapposition of stents [4]. In-stent neoatherosclerosis usually occurs early after PCI and appears to be more common after DES implantation than BMS implantation. Moreover, calcification within the in-stent neointima of patients receiving DES is characterized by calcification of fibrin, mainly observed in paclitaxel-eluting stents.

The other factors associated with early and late stent thrombosis are characteristics usually linked to coronary artery lesions, patients, and procedures. However, the specific causes of VVLST are not entirely understood. Current smoking status and total stent length >28 mm have been identified as risk factors (5). In addition, non-compliance with dual antiplatelet therapy (DAPT) may lead to thrombotic complications in the period of strut endothelization [12].

The increase in stent thrombosis seen with first-generation DES led to the intensification of DAPTs during the mid-2000s and early 2010s. Both the current European and American guidelines for DAPT duration following PCI include Class I recommendations for six months of DAPT (aspirin and clopidogrel) for stable ischemic heart disease and 12 months of DAPT [aspirin and P2Y12i, ticagrelor or prasugrel, (AHA/ACC) or prasugrel (ESC)] for those with all forms of acute coronary syndrome (ACS) [13]. A single DAPT strategy may not seem to fit all patients equally because patients have different risks of either bleeding or stent thrombosis, which determine the duration of DAPT therapies for VLST and VVLST prevention. These guidelines recommend that “it is reasonable to discontinue P2Y12 inhibitors earlier than 12 months if the risk of bleeding outweighs the anticipated benefit of continuing the second antiplatelet agent”. However, in DES implantation patients with a high risk of stent thrombosis and/or low risk of bleeding, it may be reasonable to extend DAPT beyond 12 months, and this is the preferred method to prevent stent thrombosis in DES implantation

Recently, Mori. et al (2023) reported optical coherence tomography (OCT) results from 50 VLST lesions in an Asian population with 20 years of follow-up, which revealed incidences of neoatherosclerotic rupture (44%), neointimal erosion (24%), edge disease (10%), evagination (10%), malapposition (8%), uncovered struts (2%), and in-stent calcified nodule (2%). Of note, 82% of lesions were related to neoatherosclerosis [14]. Another study on VLST involving 64 East Asian patients with a shorter follow-up of 7.5 years by Taniwaki et al. showed a lower prevalence of neoatherosclerosis (27.6%), but more strut malapposition (34.5%), uncovered struts (12.1%), in which thrombus were 8.2 times more frequent in the uncovered and malapposed struts [15]. Moreover, a study by Walse et al. among another Asian population showed that after a five-year follow-up, the incidence of VLST is similar between DES and BMS [16]. These findings suggest the likely role of neoatherosclerosis-related changes in the etiology of VLST, especially in Asian patients. Due to the lack of intravascular ultrasound (IVUS) and OCT data, we could not establish the causes of thrombus formation, which could be either due to plaque rupture or neointimal erosion. Neointimal erosion was highly suspected due to the reduction of thrombus after eight days on antithrombotic agents [17,18,19].

Our patient sustained a right ventricular STEMI due to VVLST 12.3 years after first-generation DES implantation. To our knowledge, this is the longest reported duration between implantation and stent thrombosis involving first-generation DES. Due to the unavailability of IVUS or OCT at our hospital, additional information regarding delayed endothelization, neoatherosclerosis, malposition, and fracture of the stent could not be provided. In our patient, the presumed risk factors were smoking, stent length >28 mm, and the discontinuation of aspirin and clopidogrel at 12 months after DES implantation. He had neither diabetes mellitus nor hypertension. Of note, a thrombus burden was confirmed in the patient based on a thrombotic material aspirated during thrombosuction and the thrombus decreased significantly after nine days of antithrombotic therapy. Although we cannot fully establish the pathophysiologic mechanism and risk factors for VVLST in this patient, it might be associated with the rupture of neoatherosclerotic plaque or neointimal erosion.

## Conclusions

VVLST remains a relatively rare but potentially serious complication after 12 years of DES implantation. Some of the potential associated factors include smoking, stent malapposition, uncovered struts, stent underexpansion, long stents, and neoatherosclerosis rupture. Neointimal erosion was highly suspected in our patient. To our knowledge, early discontinuation of antiplatelet therapy after PCI plays the most important role in causing VVLST. Therefore, in the absence of a high risk of bleeding, patients at increased ischemic risk may be given prolonged DAPT and life-long aspirin therapy, especially in those who underwent old-generation DES implantation.

## References

[1] Kuramitsu S, Sonoda S, Ando K (2021). Drug-eluting stent thrombosis: current and future perspectives. Cardiovasc Interv Ther.

[2] Cutlip DE, Windecker S, Mehran R (2007). Clinical end points in coronary stent trials: a case for standardized definitions. Circulation.

[3] Gupta S, Gupta MM (2008). Stent thrombosis. J Assoc Physicians India.

[4] Gupta S (2016). Very very late stent thrombosis: 9.5 years after DES implantation. Indian Heart J.

[5] Kimura T, Morimoto T, Nakagawa Y (2012). Very late stent thrombosis and late target lesion revascularization after sirolimus-eluting stent implantation: five-year outcome of the j-Cypher Registry. Circulation.

[6] Layland J, Jellis C, Whitbourn R (2009). Extremely late drug-eluting stent thrombosis: 2037 days after deployment. Cardiovasc Revasc Med.

[7] Taylor-Sutton JE, Kim MC (2011). Very late stent thrombosis approximately 7 years after deployment and one-week cessation of dual antiplatelet therapy. J Invasive Cardiol.

[8] Kaliyadan A, Siu H, Fischman DL (2014). "Very" very late stent thrombosis: acute myocardial infarction from drug-eluting stent thrombosis more than 5 years after implantation. J Invasive Cardiol.

[9] Kumar T, Shah MM, Prajapati A, Pathak S (2022). A case of "very" very late stent thrombosis: More than 12 years after DES. J Family Med Prim Care.

[10] Finn AV, Otsuka F (2012). Neoatherosclerosis: a culprit in very late stent thrombosis. Circ Cardiovasc Interv.

[11] Otsuka F, Byrne RA, Yahagi K (2015). Neoatherosclerosis: overview of histopathologic findings and implications for intravascular imaging assessment. Eur Heart J.

[12] Giustino G, Baber U, Sartori S (2015). Duration of dual antiplatelet therapy after drug-eluting stent implantation: a systematic review and meta-analysis of randomized controlled trials. J Am Coll Cardiol.

[13] Thomas A, Gitto M, Shah S (2023). Antiplatelet strategies following PCI: a review of trials informing current and future therapies. J Soc Cardiovasc Angiography Interv.

[14] Mori H, Sekimoto T, Arai T (2023). Mechanisms of very late stent thrombosis in Japanese patients as assessed by optical coherence tomography. Can J Cardiol.

[15] Taniwaki M, Radu MD, Zaugg S (2016). Mechanisms of very late drug-eluting stent thrombosis assessed by optical coherence tomography. Circulation.

[16] Walse RS, Mohanan Nair KK, Valaparambil A, Sasidharan B, Sivadasapillai H, Thulaseedharan JV (2023). Natural history of coronary stents: 14 year follow-up of drug eluting stents versus bare metal stents. Indian Heart J.

[17] Jia H, Dai J, Hou J (2017). Effective anti-thrombotic therapy without stenting: intravascular optical coherence tomography-based management in plaque erosion (the EROSION study). Eur Heart J.

[18] Hu S, Wang C, Zhe C (2017). Plaque erosion delays vascular healing after drug eluting stent implantation in patients with acute coronary syndrome: An In Vivo Optical Coherence Tomography Study. Catheter Cardiovasc Interv.

[19] Libby P (2017). Superficial erosion and the precision management of acute coronary syndromes: not one-size-fits-all. Eur Heart J.

